# Comment on “Using genomic data and machine learning to predict antibiotic resistance: A tutorial paper”

**DOI:** 10.1371/journal.pcbi.1013673

**Published:** 2025-12-01

**Authors:** Davide Chicco, Giuseppe Jurman

**Affiliations:** 1 Dipartimento di Informatica Sistemistica e Comunicazione, Università di Milano-Bicocca, Milan, Italy; 2 Institute of Health Policy Management and Evaluation, University of Toronto, Toronto, Ontario, Canada; 3 Department of Biomedical Sciences, Humanitas University, Milan, Italy; 4 Data Science for Health Unit, Fondazione Bruno Kessler, Trento, Italy; SIB: Swiss Institute of Bioinformatics, SWITZERLAND

## Abstract

A recent study by Faye Orcales and colleagues proposes a teaching curriculum on supervised machine learning applied to genomics data aimed at predicting antibiotic resistance. The article describes a traditional machine learning pipeline step-by-step in a way that is accessible to anyone, including novices. However, the authors provide a misleading piece of advice in the “Evaluating model performance” section, where they recommend that readers use accuracy and the F1 score for binary classification. We write this short formal comment on that article to reaffirm and explain why accuracy and the F1 score should be avoided in the evaluation of binary classification and why the Matthews correlation coefficient (MCC) should be employed instead. We also take this opportunity to warn readers about the dangers of *k*-fold cross-validation, which is suggested as a standard method for dividing data into training set and test set, but has several flaws and pitfalls.

## Formal comment

A recent study published by Faye Orcales et al. [1] in PLOS Computational Biology thoroughly describes a teaching proposal for a tutorial on supervised machine learning applied to genomics data for predicting antibiotic resistance. Antibiotic resistance is a significant global public health problem indeed, and computational intelligence can be an effective tool for analyzing genomics data and for identifying potential interesting data trends related to antibiotic resistance.

The article effectively outlines the datasets used, the machine learning models employed, common practices in machine learning such as cross validation, and various binary classification evaluation metrics. It also provides an overview of the contents of the six Google Colab notebooks made available by the authors to the students participating in the tutorial.

In the “Evaluating model performance” section, the authors recommend students and readers of the article calculate four metrics: accuracy, recall (true positive rate), precision (positive predictive value), and F1 score.

While we agree on the usage of recall and precision, we completely disagree with the authors regarding the F1 score and accuracy.

As we have explained in the past [2], F1 score and accuracy can be misleading when handling unbalanced datasets and, therefore, should be avoided. Instead, the Matthews correlation coefficient (MCC) should be emphasized as the most valuable metric for binary classification evaluations.

Let us suppose we have a dataset of ten elements, consisting of nine positives and one negative, and a naive classifier that always predicts 1s. The predicted values would be {1, 1, 1, 1, 1, 1, 1, 1, 1, 1}.

In this case, the accuracy would be 0.900, and the F1 score would be 0.947 (on a scale from 0 to 1), which could be interpreted as almost perfect prediction. However, if we examine the confusion matrix, we would find that we have TP (True Positives) = 9, FP (False Positives) = 1, and no TN (True Negatives) and no FN (False Negatives), indicating low quality.

The MCC, on the other hand, would be undefined in this scenario, raising a red flag for the researcher or student.

Several other similar examples could be mentioned. Consider a case where we have TP = 90, FP = 5, TN = 1, and FN = 4. Even if this classifier correctly predicts all 95 positive elements, it clearly performs poorly in predicting the negatives: only one out of five negatives is correctly identified as a true negative.

In this imbalanced case, a wise metric would yield a low result. However, accuracy would be 0.91, and the F1 score would be 0.9524 (on a scale from 0 to 1), falsely suggesting an excellent prediction. The MCC, in contrast, would be 0.135 (on a scale from -1 to +1), indicating a result similar to random chance.

Surprisingly, the authors of the [1] study are aware of this trouble with accuracy (they wrote: “For example, if 95% of the isolates in the data set are susceptible, and a model predicts that all isolates are susceptible, then the accuracy would be 95%, because the model gets it right for 95% of the cases; 95% accuracy sounds great”), but they suggest to handle it by using recall (true positive rate) together with accuracy.

We disagree and recommend anyone performing a binary classification to calculate the MCC and the four confusion matrix basic rates, such as recall (true positive rate), precision (positive predictive value) but also specificity (true negative rate) and negative predictive value.

**Fig 1:**
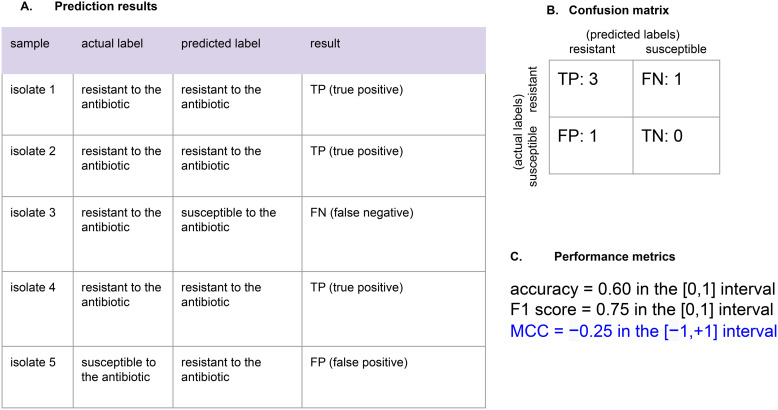
Example of how confusion matrix results are used to calculate evaluation metrics. (A) Determination of true positive (TP), true negative (TN), false positive (FP), and false negative (FN) labels. (B) The confusion matrix based on the (A) model showing total numbers of TP, TN, FP, and FN. (C) Numbers from the confusion matrix are used to calculate various evaluation metrics.

To better explain this point, we propose the example of isolate classification including the outcome measured through the Matthews correlation coefficient in Fig 1.

In that example, we have five bacterial isolates that were predicted to be resistant or susceptible to an antibiotic. We compared these five predicted labels with the original antibiotic labels of the five isolates to generate a confusion matrix having 3 TP, 1 FN, 1 FP, and no TN.

We then calculated accuracy, F1 score, and MCC for this confusion matrix, and the MCC resulted being the only informative truthful outcome of this experiment (Fig 1)

As one can notice, accuracy and F1 score produced high results, indicating overoptimistic 60% to 75% levels of correctness, respectively. The MCC, instead, produced a low negative value, clearly indicating a problem in this binary classification. The problem, in fact, is the lack of true negatives (TN). Accuracy and F1 score were unable to spot this drawback.

Even if the Matthews correlation coefficient has several advantages, we need to acknowledge that it is not a silver bullet and has some flaws, too: in particular, it can be undefined when two classes of the confusion matrix are zero [3].

Another flaw in the study is the recommendation to use cross-validation. While it is a common practice, it has several disadvantages, as the assignment of data to the *k*-folds is always arbitrary and represents only one of the possible partitions of the dataset [4].

Instead of using cross-validation, we recommend repeated hold-out validation: splitting the dataset into 80% randomly selected data elements for the training set, using the remainder for the test set, calculating the results, and then repeating this process one thousand times to report the average results. Of course, the training set /test set partition should be stratified, to keep the same general ratio of positives and negatives of the whole dataset. This approach ensures that at each iteration, a random subsample of the data is selected for the training set, making the final results more universal and generalizable than those obtained from a single short partition made through cross-validation.

The article by Faye Orcales et al. [1] also merits recognition for not mentioning and not promoting the receiver operating characteristic (ROC) curve and its area under the curve (AUC), which is a misleading index still commonly employed in machine learning studies, unfortunately [5–7].

We firmly believe that the ROC AUC should be avoided whenever possible. When ROC AUC is needed to be reported for comparison with other studies, it should be discussed thoroughly with care, highlighting its critical points.

In conclusion, we find most of the [1] article interesting and useful for students who want to learn machine learning applied to antibiotic resistance data. However, we invite the authors to advise readers and students to use the Matthews correlation coefficient to assess binary classification results, and to stay away from accuracy and F1 score in any future tutorials they might release. Moreover, we invite them to illustrate the advantages of repeated hold-out validation compared to cross-validation in their tutorial.
